# Human neural progenitors establish a diffusion barrier in the endoplasmic reticulum membrane during cell division

**DOI:** 10.1242/dev.200613

**Published:** 2022-08-04

**Authors:** Muhammad Khadeesh bin Imtiaz, Lars N. Royall, Daniel Gonzalez-Bohorquez, Sebastian Jessberger

**Affiliations:** Laboratory of Neural Plasticity, Faculties of Medicine and Science, Brain Research Institute, University of Zurich, 8057 Zurich, Switzerland

**Keywords:** Human neural progenitor, Neurogenesis, Diffusion barrier, Organoid, Cell division, ER membrane

## Abstract

Asymmetric segregation of cellular components regulates the fate and behavior of somatic stem cells. Similar to dividing budding yeast and precursor cells in *Caenorhabditis elegans*, it has been shown that mouse neural progenitors establish a diffusion barrier in the membrane of the endoplasmic reticulum (ER), which has been associated with asymmetric partitioning of damaged proteins and cellular age. However, the existence of an ER diffusion barrier in human cells remains unknown. Here, we used fluorescence loss in photobleaching (FLIP) imaging to show that human embryonic stem cell (hESC)- and induced pluripotent stem cell (iPSC)-derived neural progenitor cells establish an ER diffusion barrier during cell division. The human ER diffusion barrier is regulated via lamin-dependent mechanisms and is associated with asymmetric segregation of mono- and polyubiquitylated damaged proteins. Further, forebrain regionalized organoids derived from hESCs were used to show the establishment of an ER membrane diffusion barrier in more naturalistic tissues, mimicking early steps of human brain development. Thus, the data provided here show that human neural progenitors establish a diffusion barrier during cell division in the membrane of the ER, which may allow for asymmetric segregation of cellular components, contributing to the fate and behavior of human neural progenitor cells.

## Abstract (precis)

**Summary:** Asymmetric segregation of cellular components, which may contribute to the fate and behavior of human neural progenitors, is allowed by a diffusion barrier in the endoplasmic reticulum membrane during cell division.

## INTRODUCTION

Somatic stem cells can self-renew via symmetric, duplicating divisions or divide asymmetrically to produce daughter cells of different cell fate. Asymmetric cell division of stem cells is associated with asymmetric segregation of cellular components (Moore and Jessberger, 2017). Unequal partitioning of cell content was, for example, shown in stem cells of *Drosophila melanogaster*, in which the cell fate determinant NUMB becomes asymmetrically inherited, hence generating daughter cells with distinct fates (Rhyu et al., 1994; Cayouette and Raff, 2002). Asymmetric segregation also extends to other cell cargoes such as mitochondria, damaged proteins and centrioles (Wang et al., 2009; McFaline-Figueroa et al., 2011; Katajisto et al., 2015). Asymmetric inheritance of cargoes may mediate advantages to the stem cell, the health of which must be maintained to conserve its proliferative ability (Henderson and Gottschling, 2008). Further, asymmetrical segregation of certain cargoes is essential for the activation and function of daughter cells. For example, lysosome inheritance has been shown to correlate with the activation of hematopoietic stem cells (Loeffler et al., 2019). Moreover, under certain conditions associated with cancer and aging, proper asymmetric segregation of cargoes appears to become impaired, resulting in the disruption of tissue homeostasis and maintenance (Knoblich, 2010).

The mechanisms underlying asymmetric segregation have been studied in a variety of organisms from budding yeast, *D. melanogaster* and *Caenorhabditis elegans* to mice (Luedeke et al., 2005; Moore et al., 2015; Lee et al., 2016). Contributing to asymmetric cell division, a diffusion barrier in the membrane of the endoplasmic reticulum (ER) has been identified and proposed as a crucial mediator determining the segregation of aging factors (Shcheprova et al., 2008; Clay et al., 2014). In mouse neural progenitor cells, the ER membrane diffusion barrier becomes weakened with age, resulting in increased symmetric inheritance of damaged proteins with advancing age (Moore et al., 2015; bin Imtiaz et al., 2021). Even though a barrier in the ER membrane has been described in a variety of species, its presence and relevance for human neural progenitor cells (NPCs) remains unknown.

Using human embryonic stem cell (hESC)- and induced pluripotent stem cell (iPSC)-derived NPCs, we show that human NPCs establish a diffusion barrier in the ER membrane during cell division that is regulated via lamin-dependent mechanisms and that is associated with the asymmetric segregation of damaged proteins. Further, we used genome-edited hESC-derived forebrain organoids to show the presence of an ER membrane diffusion barrier during more naturalistic human NPC divisions.

## RESULTS AND DISCUSSION

### ER membrane diffusion barrier in human progenitors

**Fig. 1.**
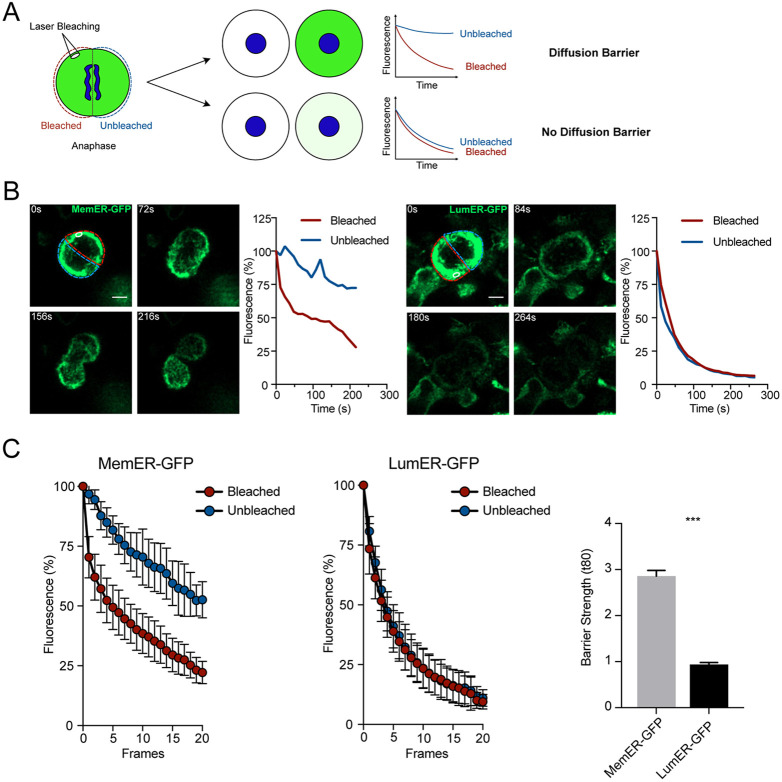
**An ER membrane diffusion barrier in human neural stem cells.** (A) Outline of the FLIP assay. A region is selected and continuously bleached in cells expressing a GFP marker. The compartment where the bleached region is located (red outline) is termed ‘Bleached’ whereas the unbleached compartment (blue outline) is termed ‘Unbleached’. The region is continuously bleached until no visible GFP signal is present between the two compartments. The fluorescence is then measured in both Bleached and Unbleached during the course of bleaching. In the presence of a diffusion barrier, loss of fluorescence is limited to Bleached (top), whereas in the absence of a diffusion barrier, loss of fluorescence is equal in both Bleached and Unbleached (bottom). (B) Single cells expressing either MemER-GFP (left) or LumER-GFP (right) undergoing FLIP assays are shown. The region bleached is circled (white). The Bleached and the Unbleached compartments of the cells are shown (red and blue, respectively). Measured fluorescence intensities for the two compartments are plotted against time for MemER-GFP (left) and LumER-GFP (right). (C) Average fluorescence intensities for the Bleached and the Unbleached regions are shown for LumER-GFP and MemER-GFP (left). Barrier strength indices of both MemER-GFP and LumER-GFP are also plotted (right) (Sel1L: *n*=16; KDEL: *n*=10). Data are mean±s.e.m. ***P<0.001 (two-tailed unpaired *t*-test). Scale bars: 5 µm.

To study whether a diffusion barrier in the ER membrane is present in human NPCs, we used fluorescence loss in photobleaching (FLIP) experiments, in analogy to experiments previously described in mouse NPCs (Fig. 1A) (Moore et al., 2015; bin Imtiaz et al., 2021). First, we developed tools to visualize the human ER membrane and lumen in human NPCs, derived from hESCs and expressing markers of NPCs such as SOX2, NES, PLZF (also known as ZBTB16) (Fig. S1A) (Costa et al., 2016). ER membrane markers used previously were toxic to human NPCs and resulted in high rates of cell death. Therefore, we tested a plethora of proteins localized to the ER membrane and eventually used Suppressor/Enhancer of Lin-12-like (SEL1L) tagged with green fluorescent protein (GFP) as a novel marker for the ER membrane (bin Imtiaz et al., 2021). Human neural progenitors derived from hESCs were transfected with fluorescent proteins targeted to either the lumen (KDEL-GFP, hereafter called LumER-GFP) or to the membrane of the ER (Sel1L-GFP, hereafter called MemER-GFP).

To probe for the existence of a diffusion barrier in human progenitor cells, we used FLIP experiments during cell division (Fig. 1A). Once cells entered anaphase, a region was selected in one compartment of the dividing cell and bleached continuously. While cells progressed through cell division, the fluorescence signal in the two compartments was measured and plotted, with the intensities normalized to the pre-bleach signal in both compartments. In the presence of a diffusion barrier limiting free movement of the tagged protein between the two compartments, the unbleached compartment will lose significantly less fluorescence signal. In the absence of a diffusion barrier, the decay in the intensity of the signal in both compartments will be equal (Fig. 1A). Fluorescence intensity of LumER-GFP showed no difference in the loss of GFP signal between the bleached and unbleached compartments, indicating that the ER is continuous and that diffusion is not limited within the lumen of the ER (Fig. 1B,C; Movie 1). In contrast, we found a localized loss of fluorescence in the bleached compartment while the unbleached compartment retained most of its GFP signal when we performed FLIP experiments using MemER-GFP (Fig. 1B,C; Movie 1). These results show the presence of a diffusion barrier within the membrane of the ER during division, limiting free diffusion of proteins in the continuous ER membrane of human NPCs (Fig. 1B,C). To corroborate the findings of an hESC-derived NPC line, we next tested a human iPSC-derived NPC line for the presence of an ER membrane diffusion barrier (Hruska-Plochan et al., 2021 preprint). Again, we found that iPSC-derived NPCs, expressing SOX2, NES and PLZF, established a diffusion barrier in the ER membrane during anaphase (Fig. S1B-D).

These results show that human NPCs establish a diffusion barrier that restricts free diffusion of ER membrane proteins during mitosis. Similar mitotic ER membrane diffusion barriers have been reported in yeast, mice and *C. elegans*, and our results suggest that this is a conserved mechanism between stem cells of different species (Luedeke et al., 2005; Moore et al., 2015; Lee et al., 2016).

### Progerin regulates human ER barrier strength

**Fig. 2.**
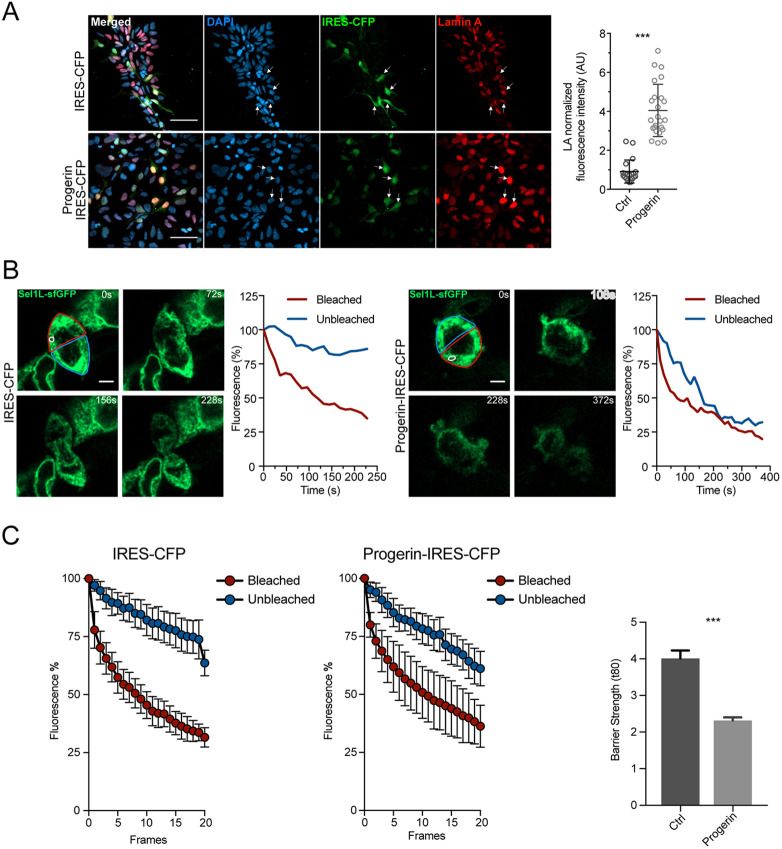
**Progerin expression weakens the ER membrane diffusion barrier.** (A) Representative images (left) and quantification (right) showing the expression of progerin in human NPCs. Human NPCs were transduced with either a control (IRES-CFP) or progerin (Progerin IRES-CFP) plasmid and were then stained against DAPI (blue), IRES-CFP (green) and lamin-A (red). (B) Single cells expressing MemER-GFP and either control (IRES-CFP; right) or progerin (Progerin-IRES-CFP; left) undergoing FLIP assays are shown. The region bleached is circled (white). The Bleached and the Unbleached compartments of the cells are shown (red and blue, respectively). Measured fluorescence intensities for the two compartments are plotted against time (right). (C) Average fluorescence intensities for cells expressing either control (IRES-CFP) or progerin (Progerin-IRES-CFP) that underwent FLIP assays are shown. Bleached (red) and Unbleached (blue) fluorescence intensity averages are shown over time (s; left). Barrier strength indices of control and progerin cells are also plotted (right) (Ctrl: *n*=15; Progerin: *n*=15). Data are mean±s.e.m. ***P<0.001 (two-tailed unpaired *t*-test). Scale bars: 50 µm (A); 5 µm (B).

Next, we aimed to modulate the strength of the human ER membrane diffusion barrier. Lamins, which are intermediate filaments in the nucleus, have been implicated in the weakening of the diffusion barrier that occurs with advancing age in mouse NPCs (bin Imtiaz et al., 2021). Progerin, which is a mutant form of lamin A, implicated in Hutchinson-Gilford progeroid syndrome (HGPS), has been previously used to mimic certain cellular aging phenotypes *in vitro* (Miller et al., 2013; Moore et al., 2015). To determine whether the human ER membrane diffusion barrier is regulated by progerin, we overexpressed progerin in human NPCs (Fig. 2A). We then performed FLIP assays on MemER-GFP human NPCs and observed that progerin expression weakened the strength of the ER membrane diffusion barrier in human NPCs (Fig. 2B,C; Movie 2), suggesting that, similar to previously obtained data in mouse NPCs, progerin overexpression affects the strength of the ER membrane diffusion barrier in human NPCs.

### Asymmetric segregation of damaged proteins

**Fig. 3.**
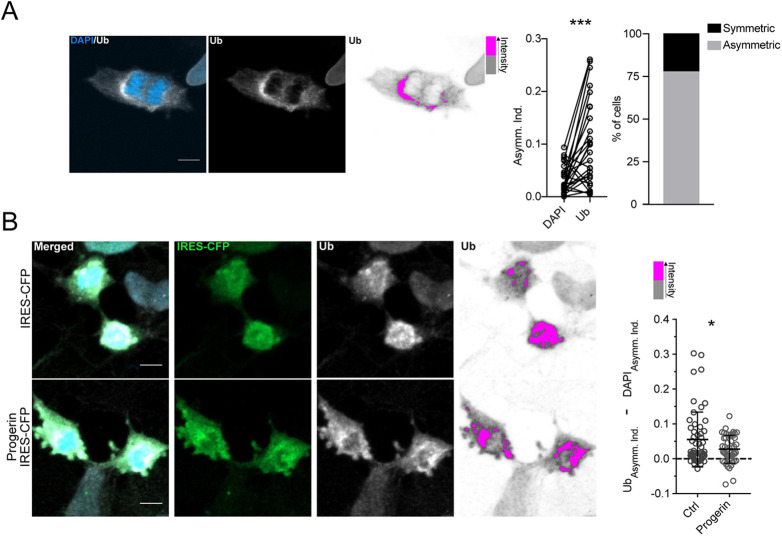
**Ubiquitylated proteins are asymmetrically segregated in human NPCs.** (A) A representative image showing asymmetrical segregation of ubiquitylated proteins. The human NPCs were stained for DAPI (blue) and ubiquitin (Ub; white; left). High ubiquitin intensity in this example is shown in pink whereas low ubiquitin intensity is shown in grey. Asymmetry indices of DAPI and ubiquitin were then calculated for each cell and plotted (middle). Cells with a higher asymmetry index of ubiquitin were classified as asymmetric and plotted (*n*=24; right). (B) Representative images of human NPCs expressing either control (IRES-CFP) or progerin (Progerin-IRES-CFP) showing asymmetric segregation of ubiquitylated proteins. Human NPCs were stained for DAPI (blue), IRES-CFP (green) and ubiquitin (white). High ubiquitin intensity is shown in pink whereas low ubiquitin intensity is shown in grey. The difference between asymmetry indices of ubiquitin and asymmetry indices of DAPI is plotted (Ctrl: *n*=56; Progerin: *n*=59). Data are mean±s.e.m. *P<0.05, ***P<0.001 (two-tailed unpaired *t*-test). Scale bars: 5 µm.

Previous studies suggested a correlation between the strength of the ER membrane diffusion barrier and asymmetric segregation of mono- and polyubiquitylated proteins, as an indirect measure of levels of damaged proteins, even though ubiquitylation is also involved in a plethora of biological processes independent of protein damage (Komander and Rape, 2012; Clay et al., 2014; Moore et al., 2015). Thus, we next tested whether ubiquitylated proteins are asymmetrically segregated during human NPC divisions. Using immunostaining to label mono- and polyubiquitylated proteins, we found that human NPCs showed asymmetric segregation of ubiquitylated proteins (Fig. 3A). We next analyzed whether a weakened ER membrane diffusion barrier, induced by overexpression of progerin, affects asymmetric segregation of ubiquitylated proteins. Indeed, progerin-expressing cells, associated with a weakened ER membrane diffusion barrier, showed increased symmetric segregation of ubiquitylated proteins compared with control cells (Fig. 3B), suggesting that the ER membrane diffusion barrier is involved in the asymmetric segregation of ubiquitylated proteins in human NPCs.

### ER membrane diffusion barrier in neural progenitors of regionalized forebrain organoids

**Fig. 4.**
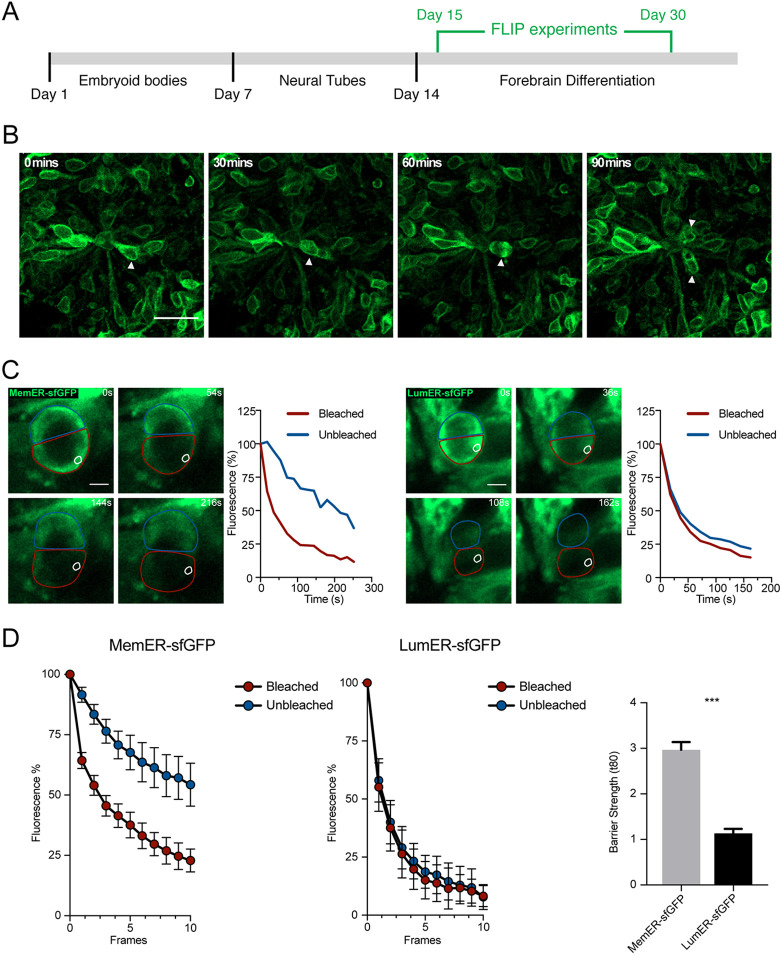
**An ER membrane diffusion barrier is established in dividing neural progenitors of human forebrain organoids.** (A) The experimental outline of the FLIP assays on forebrain organoids is shown. Embryoid bodies from hESCs, which stably expressed either the LumER-GFP or MemER-GFP, were made. This was followed by induction of neural tubes and then organoids mimicking human forebrain differentiation. FLIP assays were performed between 15 and 30 days post formation of embryoid bodies. (B) A neural progenitor division in a MemER-GFP organoid is shown. The neural progenitor (arrowhead; 0, 30, 60 min) performs interkinetic nuclear migration towards the ventricular unit before undergoing mitosis. The two daughter cells are also shown (arrowheads; 90 min). (C) Single cell traces of LumER-GFP and MemER-GFP progenitors undergoing mitosis in organoids. An area (white circle) was selected and bleached continuously from the onset of anaphase until no GFP signal was visible between the two compartments. The fluorescence intensities of the bleached (red) and unbleached (blue) regions are plotted (right) for each cell. (D) Average fluorescence intensities of the two compartments for both LumER-GFP and MemER-GFP cells are plotted against frames (left). Barrier strength indices for LumER-GFP and MemER-GFP are also plotted (right) (Sel1L: *n*=16; KDEL: *n*=8). Data are mean±s.e.m. ***P<0.001 (two-tailed unpaired *t*-test). Scale bars: 20 µm (B); 5 µm (C).

Following our *in vitro* studies on two-dimensional (2D) cultured cells, we next aimed to study whether human NPCs establish a diffusion barrier in more naturalistic three-dimensional (3D) *in vitro* models of the developing human brain, i.e., regionalized forebrain organoids. To obtain forebrain organoids allowing for FLIP-based experiments, we first established hESC lines that constitutively expressed either MemER-GFP or LumER-GFP from the human safe harbor site AAVS1 (Chen et al., 2015). We mixed hESCs positive for MemER-GFP or LumER-GFP with non-labeled hESCs to produce mosaic human forebrain organoids that enabled FLIP to be performed on individual cells of the densely packed ventricular zone, consisting largely of SOX2-expression NPCs that orient themselves around ventricle-like structures within cortical units (Fig. 4A; Fig. S2A; Qian et al., 2016; Denoth-Lippuner et al., 2021). We observed a mosaic distribution of GFP-positive cells throughout the organoids, indicating that the constitutive expression of the tagged markers did not confer a disadvantage to labeled cells. We directly observed cell division events of NPCs in organoids using time-lapse imaging over 4 h (Fig. 4A,B).

We then performed FLIP assays of dividing NPCs in LumER-GFP and MemER-GFP-labeled organoids (Fig. 4C; Movie 3). In analogy to 2D-cultured NPCs, we found that LumER-GFP cells displayed equal loss of fluorescence in the two compartments during division, indicating that the ER membrane is continuous and that LumER-GFP diffuses freely between the two compartments (Fig. 4C,D). However, cells expressing MemER-GFP displayed reduced fluorescence loss in the unbleached compared with the bleached compartment, clearly indicating the establishment of an ER membrane diffusion within dividing NPCs in hESC-derived organoids (Fig. 4C,D). When the unbleached and bleached fluorescence intensities were subtracted for individual cells, we observed an ER diffusion barrier in the majority of cells expressing MemER-GFP (Fig. S2B,C). Thus, our results identify a diffusion barrier in the ER membrane established in human NPCs during cell divisions. However, at this time it remains unknown whether the ER diffusion barrier is indeed required for proper asymmetric cell divisions. Furthermore, the fate of cells establishing a barrier in the ER membrane will have to be analyzed in future experiments, given the current technical difficulty to combine FLIP approaches with imaging-based lineage tracing in hESC-derived brain organoids.

The results shown here demonstrate the presence of an ER membrane diffusion barrier during human NPC divisions. Similar diffusion barriers in the membrane of the ER have been reported previously in yeast, mouse and *C. elegans* cells (Luedeke et al., 2005; Moore et al., 2015; Saarikangas and Barral, 2015; Lee et al., 2016). Our results suggest that the establishment of an ER membrane diffusion barrier represents a conserved mechanism between stem cells of different species. A weakening of the ER barrier with progerin overexpression suggests that progerin acts as regulator of the barrier strength, as previously described in mouse cells (Moore et al., 2015; bin Imtiaz et al., 2021). However, the molecular identity of the ER barrier in mammalian cells remains poorly understood. This is in contrast to budding yeast, in which substantial progress has been made over recent years to characterize the molecular composition and functional properties of diffusion barriers in the ER membrane during cell divisions (Clay et al., 2014; Megyeri et al., 2019; Prasad et al., 2020). In human NPCs, the specific cargoes that the barrier in the ER membrane partitions remain largely unknown, even though our data found a correlation between asymmetric segregation of mono- and polyubiquitylated proteins with barrier strength. The data presented here identify that human NPCs establish a diffusion barrier in the ER membrane during cell division. Future studies will need to identify whether disruption or weakening of the ER barrier affects human NPC behavior and early steps of human brain development. Given previous work in budding yeast, *C. elegans* and mouse progenitor cells, our finding that human progenitors establish an ER diffusion barrier is not unexpected. However, we provide direct experimental evidence of an ER diffusion barrier in human progenitors using different lines of pluripotent cell-derived NPCs and brain organoids combined with FLIP imaging. Thus, the data shown here represent the foundation for future experiments with the aim of furthering our understanding of the role of asymmetric segregation during human progenitor cell divisions.

## MATERIALS AND METHODS

### Two-dimensional cell culture and forebrain organoids

Both lines of human NPCs were plated on Poly-L-Ornithine (Sigma-Aldrich) and Laminin (Sigma-Aldrich) -coated plates. The media for human NPCs derived from hESCs (Costa et al., 2016) was supplemented with a B27-supplement minus vitamin A (Thermo Fisher Scientific), N2-supplement (Thermo Fisher Scientific), Neurobasal (Thermo Fisher Scientific), EGF-2 (PeproTech), FGF (PeproTech) and DMEM/F-12 GlutaMAX (Thermo Fisher Scientific). The human NPCs derived from iPSCs [a gift from Polymenidou group (University of Zurich, Switzerland; Hruska-Plochan et al., 2021 preprint)], were plated in media supplemented with DMEM/F12 (Thermo Fisher Scientific), 0.5× B27-supplement (Thermo Fisher Scientific), 0.5× N2 supplement (Thermo Fisher Scientific), 1× GlutaMAX (Thermo Fisher Scientific) and 20 ng/ml bFGF (Gibco, #PHG0261). Both lines were passaged with 0.05% Trypsin (Thermo Fisher Scientific) that was then blocked with an equal volume of Defined Trypsin Inhibitor (Thermo Fisher Scientific). The media was changed every 2 days. Human NPCs were counted and 4×10^6^ cells were used for electroporations. Cells were then resuspended in 100 µl nucleofactor solution (Lonza) that contained 3 µg of the plasmids. The cells were then electroporated using the AMAXA electroporation system (Lonza) using the A-033 program. The cells were then plated in Poly-Ornithine (Thermo Fisher Scientific) and Laminin (Thermo Fisher Scientific) -coated plates.

H9 hESCs were maintained in feeder-free conditions without antibiotics in TeSR-E5 Plus (Stem Cell Technologies, 05825) on hESC qualified Matrigel (Corning, 354277) -coated plates. Routine passaging was performed with ReLeSR™ (Stem Cell Technologies, 05872), and 10 µM Y-27632 (Stem Cell Technologies, 72302) was added to media post passaging. For long-term storage, hESCs were stored at −170°C in CryoStore^®^ CS10 (Sigma-Aldrich, C2874). All experiments with hESCs received approval from the Canton of Zurich Kantonale Ethik-Kommision (KEK). Electroporation of hESCs was conducted with an Amaxa Nucleofector II using program A-23 according to the manufacturer's guidelines. Before electroporation, hESCs were treated with mTeSR™ Plus containing 10 µM Y-27632 for at least 2 h. Two million cells were passaged with Accutase (Sigma-Aldrich, A6964) and were resuspended in Nucleofector V (Lonza, VCA-1003). Positive cells were selected for Neomycin resistance using 100 µg/ml G418 sulfate (Gibco, 10131035). Organoids were produced with a mix of wild-type and GFP-expressing hESCs at a ratio of 1:1 in order to reduce background fluorescence. Human forebrain organoids were generated via an adapted protocol (Qian et al., 2018). Alterations were: hESCs were grown in feeder-free conditions and at day 0 were passaged to get single cells. Single cells were aggregated in AggreWell™800 (Stem Cell Technologies, 34811) following the manufacturer's instructions. Embryoid bodies were harvested the following day and maintained in mTeSR™-E5 (Stem Cell Technologies, 05916) with 2 µM Dorsomorphin (Sigma-Aldrich, P5499) and 2 µM A83-01 (Tocris, 2939). From day 4 we followed the protocol described in Qian et al. (2018). Organoids were fixed for 30 min in 4% paraformaldehyde (PFA) and were stored in 30% sucrose until further use.

### Cloning and constructs

Sel1L sequence (corresponding to amino acids 178-310 from AAH57452.1; gift from M. Molinari, Institute for Research in Biomedicine, Bellinzona, Switzerland) and KDEL was cloned into a retroviral GFP-expressing vector (Addgene plasmid #16664). Progerin construct (Addgene plasmid #17663) was cloned into a retroviral backbone vector that contained a CAG promoter followed by an internal ribosome entry site (IRES) and CFP. For control plasmid, the same plasmid without progerin cloned in was used. Flpe-ERT2 was removed from AAVS1-Neo-CAG-Flpe-ERT2 (Addgene plasmid #68460) via restriction digest and replaced with Se1L-GFP or KDEL-GFP.

### Retrovirus transduction

We plated 90,000 human NPCs in 12-well plates coated with Poly-L-Ornithine (Sigma-Aldrich) and Laminin (Sigma Aldrich) and incubated at 37°C overnight. Next day, the media was changed in the morning. Six hours after the media change, 500 µl of the media was transferred to a tube and 1 µl of the corresponding virus was added.

### Image acquisition

Zeiss LSM800 were used to acquire the images. ImageJ/Fiji was used to quantify all the images. The images were blinded before the counting/analysis was performed.

### FLIP assays

We plated 90,000 human NPCs in a well of Chambered Coverglass wells that had been coated with Poly-L-Ornithine (Sigma-Aldrich) and Laminin (Sigma-Aldrich). Then a 63× oil-immersive objective was used to carry out the following imaging. For human NPCs, the wells were manually scanned to identify GFP-positive cells in mitosis. Upon identification, the cells were marked and once they started undergoing anaphase, a region of interest was selected close to the future cleavage furrow. A prebleach image was taken before a bleach with 80 iterations and 3% laser power was applied. This bleaching was applied over 8 s and the next image was then acquired. There was a 12 s interval between each subsequent image, with 8 s being used for the bleach. The cell was continuously bleached in this manner until no GFP signal was visible between the two compartments of the dividing cell. Then, the fluorescence intensities in the bleached and the unbleached compartments were measured. The fluorescent intensities for both compartments was then divided by the area. The background intensities were subtracted from both bleached and unbleached intensities. All the measured intensities were normalized to the pre-bleach image that had been set to 100%. For the average intensity graphs, fluorescent intensities in both compartments was averaged across all cells (Figs 1C, 3C; Fig. S1B).

Organoids were embedded into Phenol Red-free growth-factor-reduced Matrigel (Corning) that was allowed to polymerize for 30 min at 37°C, onto chambered coverglasses. Media was then added before performing FLIP assays. For FLIP on the organoids, a 20× objective with a digital zoom of 2× was used. The positions of cortical units were marked and scanned manually in the GFP channel to identify cells undergoing mitosis. Once anaphase commenced in the identified cells, a prebleach picture was acquired. This was followed by selecting and bleaching a region close to the future cleavage furrow. The bleach was performed with 80 iterations and 3% laser power. The time interval between images was set at 18 s, where 12 s were needed to perform the required bleaching. The cells were continuously bleached until there was no GFP signal visible between the two compartments of the dividing cell. Then, the fluorescence intensities in the bleached and the unbleached compartments were measured. The fluorescence intensities for both compartments were then divided by the area. The background intensities were subtracted from both bleached and unbleached intensities. All the measured intensities were normalized to the pre-bleach image that had been set to 100%. For the average intensity graphs, fluorescence intensities in both compartments was averaged across all cells (Fig. 4D).

The barrier strengths were calculated by first plotting the fluorescence intensities from both the bleached and unbleached compartments in PRISM. These were then each fitted to a one-phase decay with the following constraints: Plateau=0, Y0=100, and K>0. The value of the constant K was determined from the fit in Prism and substituted into the following equation to calculate the time it takes for the corresponding compartment to fall to 80% from the initial 100% of fluorescence signal.

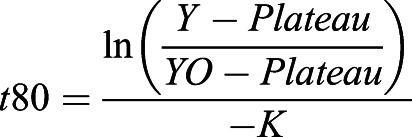
In this, Y=80, Y0=100, Plateau=0 and K was derived from the corresponding fit in Prism.

To calculate the barrier strengths, the *t*80 for unbleached was divided by *t*80 for bleached.

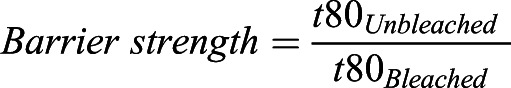
To calculate the standard error in each compartment, the following equation was used.

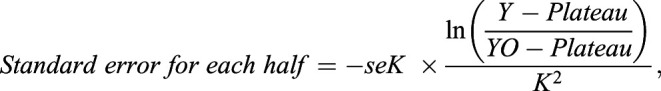
where seK is the standard error of the constant K, derived from Prism.

For the total standard error, the following equations were used.

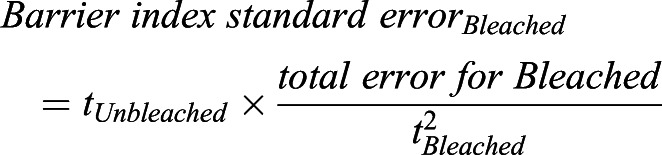


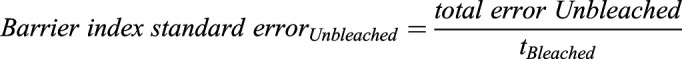


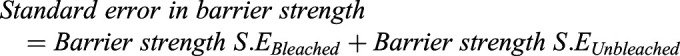


This analysis has been previously described (bin Imtiaz et al., 2021).

### Immunostaining

For immunostaining, coverslips were first coated with Poly-L-Ornithine (Sigma-Aldrich) for 1 h at 37°C and then overnight with Laminin (Sigma Aldrich) at 4°C. We plated 90,000 human NPCs on the coated coverslips. They were then fixed with 4% PFA (Sigma-Aldrich) for 15 min at room temperature before being washed three times with PBS. After fixation, the cells were blocked and permeabilized with 3% donkey serum (Millipore) and 0.20% Triton X-100 (Sigma-Aldrich) in Tris buffered saline (TBS) for 30 min at room temperature. They were then stained with different combinations of the following primary antibodies: goat α-GFP (1:1000, Rockland, 600-101-215), chicken α-GFP (1:100, Aves, GFP.1020), mouse α-Lamin A (1:1000, Millipore, MAB3540), mouse α-mono- and polyubiquitin (1:100, Enzo Life Sciences, BML-PW8810-0500), rabbit α-PLZF (1:100, Santa Cruz Biotechnology, sc22839), mouse α-PH3 (1:250, Abcam, ab14955), rabbit α-SOX2 (1:200, Millipore, ab5603), rat α-KI67 (1:500, E-biosciences, 14-5698-82), mouse α-nestin (1:200, Millipore, MAB5326), chicken α-GFAP (1:500, Novus, NBP1-05198). The primary antibodies were incubated overnight at 4°C in the blocking buffer. The following day, the cells were washed three times with TBS. Then, the cells were incubated with the appropriate secondary antibodies [donkey anti-rabbit (711-005-152), anti-goat (705-005-147), anti-mouse (115-005-003) and anti-chicken (703-005-155); all from Jackson ImmunoResearch] coupled to different fluorophores for 2 h at room temperature in the blocking buffer. Following this, the cells were washed three times with TBS and stained against DAPI. The coverslips were then mounted with ImmuMount (Thermo Fisher Scientific) and imaged.

### Statistical tests

For statistical analyses two-tailed unpaired *t*-tests were performed. Statistical tests were performed using Prism. The graphs plotted show mean±s.e.m. unless otherwise stated. The *P*-values are denoted by the following: ns>0.05, *P<0.05, **P<0.01, ***P<0.001.

## Supplementary Material

Supplementary information

Reviewer comments
