# Supplementary material for: Human neural progenitors establish a diffusion barrier in the endoplasmic reticulum membrane during cell division
Source: Development. 2022 Aug 4;149(20):dev200613. doi: 10.1242/dev.200613 (PMC9440750; doi:10.1242/dev.200613)
Supplement: Supplementary information [file develop-149-200613-s1.pdf]

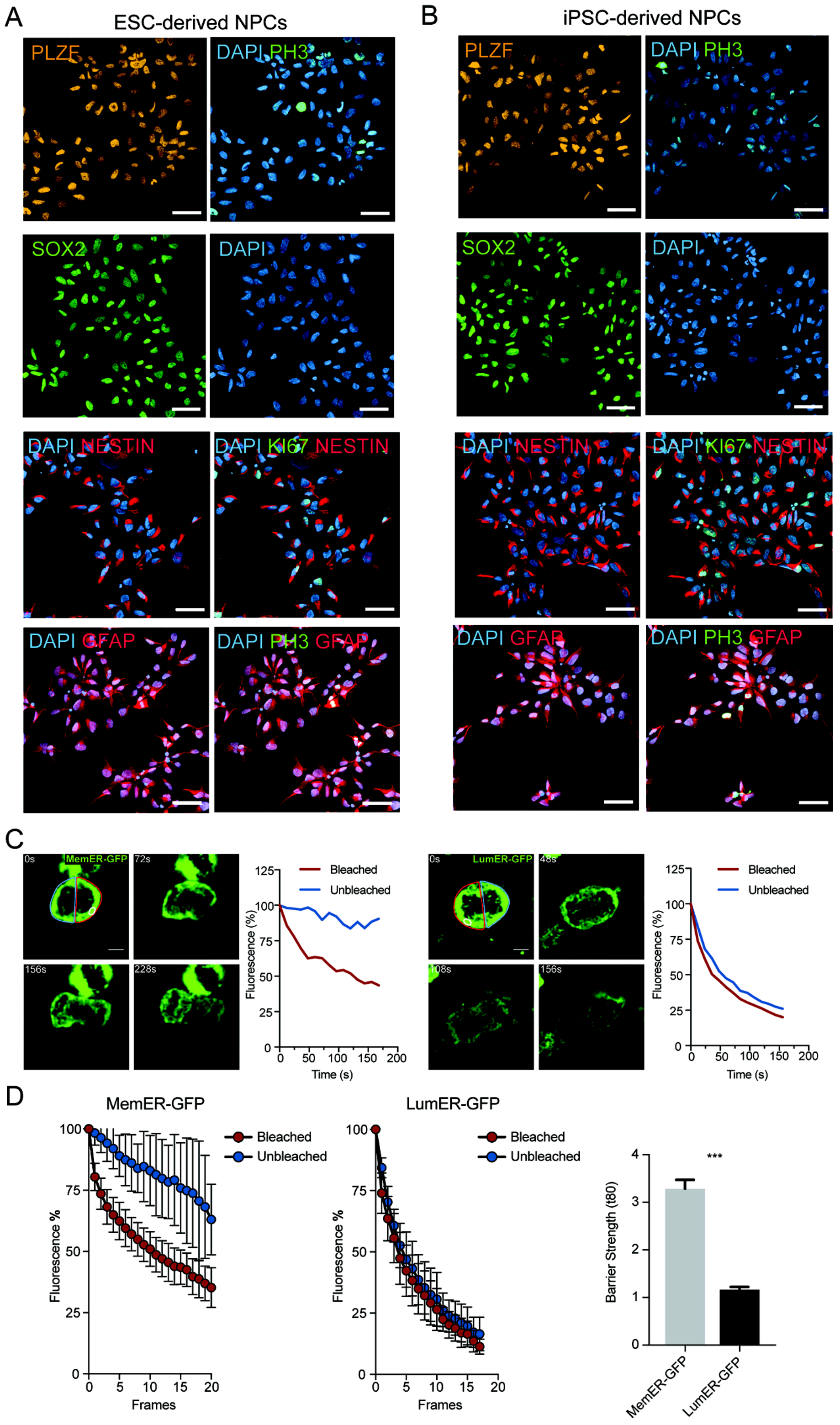
A
ESC-derived NPCs
PLZF
DAPI PH3
SOX2
DAPI
DAPI NESTIN
DAPI KI67 NESTIN
DAPI GFAP
DAPI PH3 GFAP
B
iPSC-derived NPCs
C
0s
MemER-GFP
72s
156s
228s
Bleached
Unbleached
Fluorescence (%)
Time (s)
LumER-GFP
48s
108s
D
MemER-GFP
LumER-GFP
Fluorescence %
Frames
Barrier Strength (t80)
***

Fig. S1. An ER membrane diffusion barrier is established in hESC- and iPSC-derived NPCs.

(A) Human ESC-derived NPCs express previously described marker proteins such as PZLF, SOX2, GFAP, NESTIN (as indicated in the panels) and are proliferative, as measured with PH3 and KI67. Additional characterizations of the ESC-derived NPC line have been published before (Costa et al., 2016).

(B) Human iPSC-derived NPCs express previously described marker proteins such as PZLF, SOX2, GFAP, NESTIN (as indicated in the panels) and are proliferative, as measured with PH3 and KI67. Additional characterizations of the iPSC-derived NPC line have been published before (Hruska-Plochan et al., 2021).

(C) Single cells expressing either LumER-GFP (right) or MemER-GFP (left) undergoing FLIP assays are shown. The region bleached is highlighted (white). The Bleached and the Unbleached compartments of the cells are shown (red and blue respectively). Measured fluorescence intensities for the two compartments are plotted against time (right).

(D) Average fluorescence intensities for the Bleached and the Unbleached regions are shown for LumER-GFP and MemER-GFP (left). Barrier strength indices of both MemER-GFP and LumER-GFP are also plotted (right). Sel1L: n = 21; KDEL: n = 9.

Scale bars represent 50µm (A, B) and 5µm (C). ***$p < 0.001$

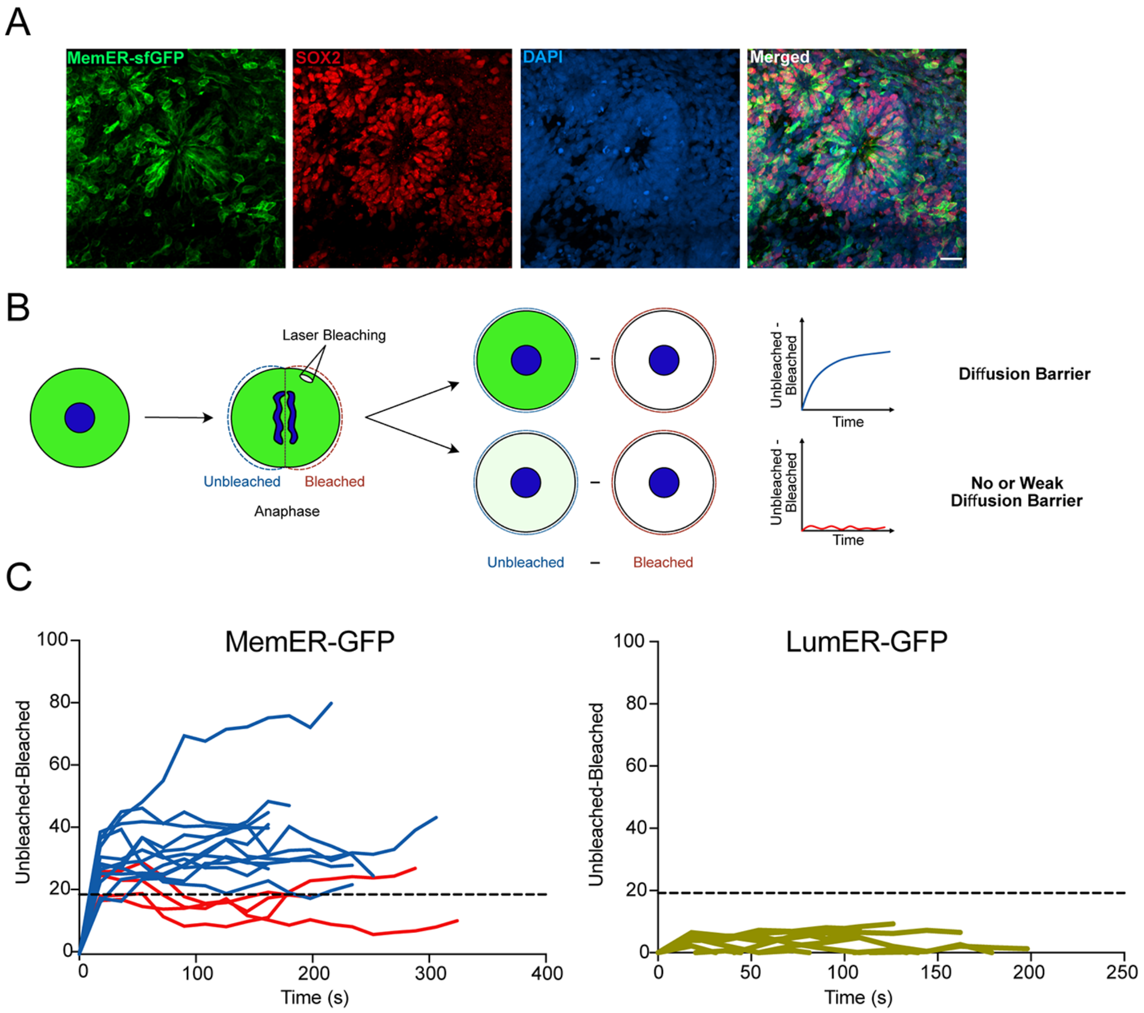

Fig. S2. Neural progenitors in human forebrain organoids establish an ER membrane diffusion barrier. Related to Figure 4.

(A) Human forebrain organoids stained against neural progenitor markers are shown. DAPI signal (blue), MemER-GFP (green) and SOX2 (red) is shown.

(B) Outline for the difference analysis is shown. The cell undergoes FLIP as described before. Then, the bleached intensity is subtracted from the unbleached intensity at each timepoint. In the presence of a diffusion barrier, the difference between the unbleached and bleached would be high (blue)

whereas in the absence of a barrier, the difference between the two will be low (red).

(C) Difference analysis for each cell in the human forebrain organoids that had underwent FLIP assays is shown. For Lum-ER-GFP, all cells showed a weak or no diffusion barrier (red). For MemER-GFP, 4 cells showed a weak or no diffusion (red) barrier whereas 12 cells showed a diffusion barrier (blue). The dotted line above cells were classified as having a barrier is at y=19.18, which is the standard deviation of the LumER-GFP averaged over all the timepoints and multiplied by 6. Scale bars represent 20μm (A).

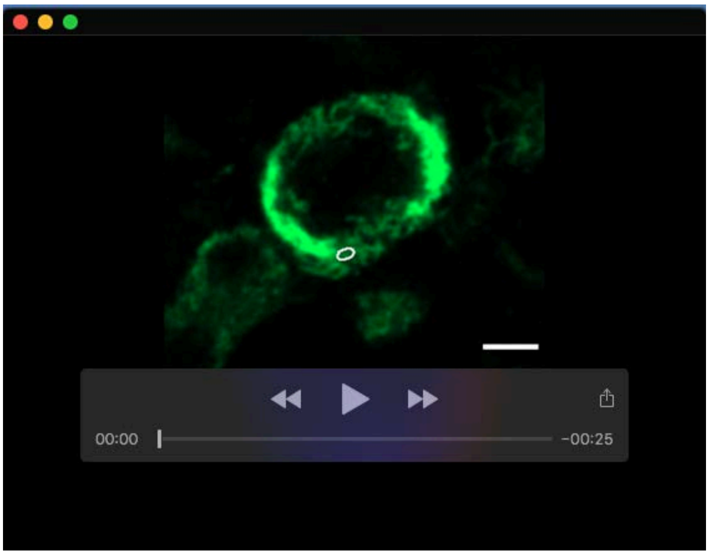

Movie 1. FLIP experiments on human NPCs expressing either LumER-GFP or MemER-GFP are shown. The human NPCs were derived from either human iPSCs or human ESCs, as indicated in the movie. Bleached area (white region) is shown exactly in the first frame and approximated in the subsequent frames. Scale bar represents 5μm.

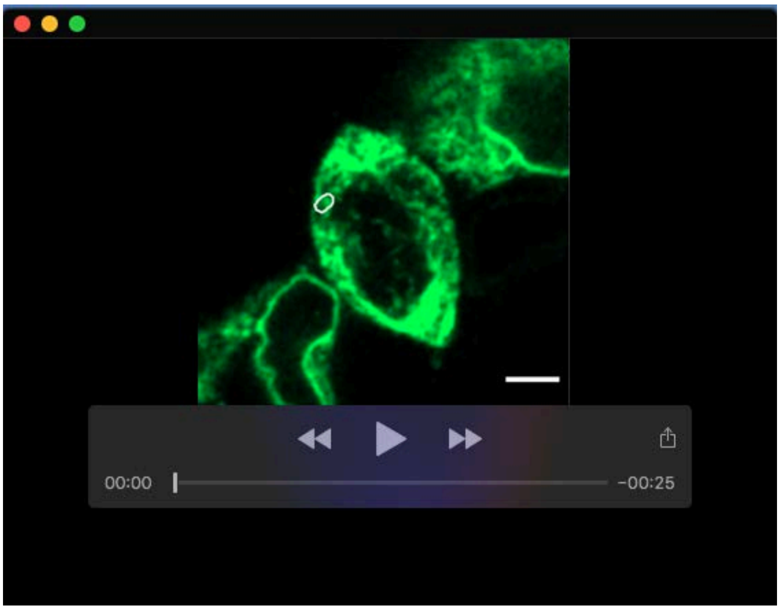

Movie 2. FLIP experiments on human NPCs expressing MemER-GFP with either IRES-CFP (control) or Progerin-IRES-CFP (Progerin) are shown. The NPCs were derived from human ESCs. Bleached area (white region) is shown exactly in the first frame and approximated in the subsequent frames. Scale bar represents 5μm.

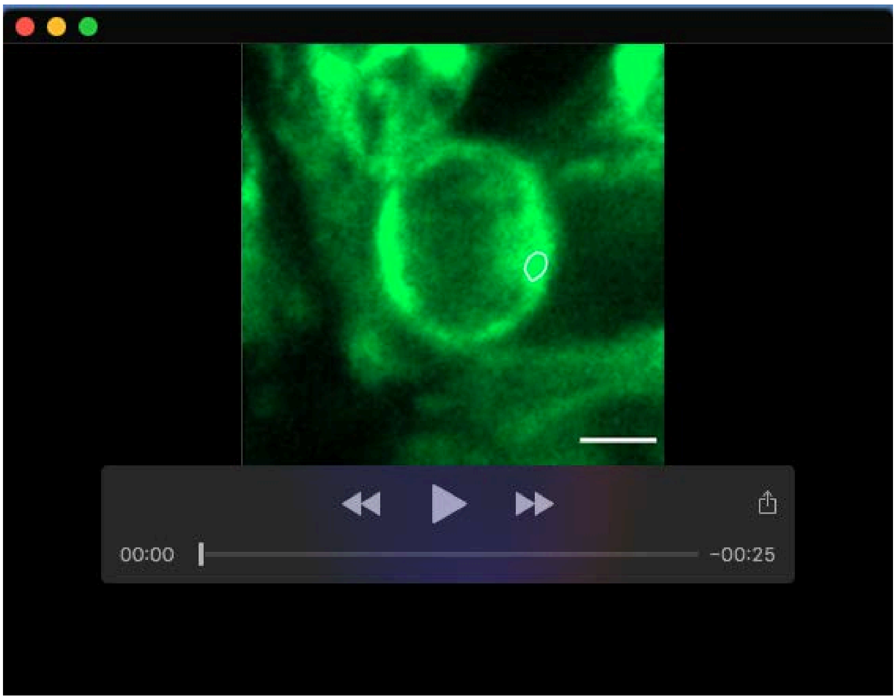

Movie 3. FLIP experiments on human forebrain organoids expressing LumER-GFP or MemER-GFP are shown. Bleached area (white region) is shown exactly in the first frame and approximated in the subsequent frames. Scale bar represents 5µm.

Supplementary References

Costa, V., Aigner, S., Vukcevic, M., Sauter, E., Behr, K., Ebeling, M., Dunkley, T., Friedlein, A., Zoffmann, S., Meyer, C. A. et al. (2016) 'mTORC1 Inhibition Corrects Neurodevelopmental and Synaptic Alterations in a Human Stem Cell Model of Tuberous Sclerosis', *Cell Rep* 15(1): 86-95.

Hruska-Plochan, Marian, Betz, Katharina M., Ronchi, Silvia, Wiersma, Vera I., Maniecka, Zuzanna, Hock, Eva-Maria, Laferriere, Florent, Sahadevan, Sonu, Hoop, Vanessa, Delvendahl, Igor et al. (2021) 'Human neural networks with sparse TDP-43 pathology reveal NPTX2 misregulation in ALS/FTLD', *bioRXiv*: 2021.12.08.471089.

Development • Supplementary information
