# Supplementary material for: Human neural progenitors establish a diffusion barrier in the endoplasmic reticulum membrane during cell division
Source: Development. 2022 Aug 4;149(20):dev200613. doi: 10.1242/dev.200613 (PMC9440750; doi:10.1242/dev.200613)
Supplement: Reviewer comments [file develop-149-200613_review_history.pdf]

Development

Human neural progenitors establish a diffusion barrier in the endoplasmic reticulum membrane during cell division

Muhammad Khadeesh bin Imtiaz, Lars N. Royall, Daniel Gonzalez-Bohorquez and Sebastian Jessberger

DOI: 10.1242/dev.200613

Editor: Matthias Lutolf

Review timeline

Original submission: 2 February 2022
Editorial decision: 18 March 2022
First revision received: 8 June 2022
Accepted: 20 June 2022

Original submission

First decision letter

MS ID#: DEVELOP/2022/200613

MS TITLE: Human neural progenitors establish a diffusion barrier in the ER membrane during cell division

AUTHORS: Khadeesh Bin Imtiaz, Lars N Royall, and Sebastian Jessberger

I have now received all the referees' reports on the above manuscript, and have reached a decision. The referees' comments are appended below, or you can access them online: please go to BenchPress and click on the 'Manuscripts with Decisions' queue in the Author Area.

As you will see, the referees express considerable interest in your work, but have some significant criticisms and recommend a substantial revision of your manuscript before we can consider publication. If you are able to revise the manuscript along the lines suggested, which may involve further experiments, I will be happy receive a revised version of the manuscript. Your revised paper will be re-reviewed by one or more of the original referees, and acceptance of your manuscript will depend on your addressing satisfactorily the reviewers' major concerns. Please also note that Development will normally permit only one round of major revision. If it would be helpful, you are welcome to contact us to discuss your revision in greater detail. Please send us a point-by-point response indicating your plans for addressing the referee’s comments, and we will look over this and provide further guidance.

Please attend to all of the reviewers' comments and ensure that you clearly highlight all changes made in the revised manuscript. Please avoid using 'Tracked changes' in Word files as these are lost in PDF conversion. I should be grateful if you would also provide a point-by-point response detailing how you have dealt with the points raised by the reviewers in the 'Response to Reviewers' box. If you do not agree with any of their criticisms or suggestions please explain clearly why this is so.

Reviewer 1

Advance summary and potential significance to field

In this paper, bin Imtiaz et al. describe the diffusion barrier in the endoplasmic reticulum of dividing human neuronal progenitor cells. They show that this diffusion barrier does exist in the

© 2022. Published by The Company of Biologists under the terms of the Creative Commons Attribution License (https://creativecommons.org/licenses/by/4.0/).

human context, similarly to earlier findings in yeast or mouse neuronal progenitors. They also show that this barrier causes asymmetric segregation of poly-ubiquitinated proteins, and that it is increased upon overexpression of progerin, a mutant form of LaminB1.

Comments for the author

Overall the study is well done and the results are clean. My main criticism is the limited advances that it provides. It is expected that the findings made in mouse neuronal progenitors, for such a fundamental process, will be confirmed in human neuronal progenitors. While this constitutes a nice confirmation, it provides little advance to our understanding of asymmetric divisions. The authors could take advantage of their system to perform a more quantitative study on this barrier in a more progenitor-type specific manner.

Major point:
- There is no data showing that the authors are actually looking at neuronal progenitors, and also there is no information on the nature of this progenitors. Are we looking at neuroectoderm, radial glia or other types of progenitors ?

Minor point:
- Some references cited in the tect do not appear in the reference list (for example bin Imtiaz et al. 2021)

<u>Reviewer 2</u>

Advance summary and potential significance to field

The study by bin Imtiaz et al., corroborates previous work showing that mouse neural progenitors establish a diffusion barrier in the membrane of the endoplasmic reticulum (ER) by showing the existence of an ER-diffusion barrier in human neural progenitor cells. Here using human ESC and iPSC derived neural progenitors in 2D and 3D cultures, the authors use FLIP imaging to show that some dividing human neural progenitors: (1) establish an ER diffusion barrier, (2) this ER diffusion barrier is weakened by Progerin overexpression and (3) there is asymmetric segregation of ubiquitinated proteins which is diminished with Progerin overexpression.

Comments for the author

The work appears to be technically sound with use of solid assays and methodology and appropriate controls. However, the work is correlative and does not show a causal relationship between the establishment of the ER diffusion barrier and the asymmetric segregation of damaged proteins, underlying the specification of fate and behavior of human neural progenitor cells. With that said, I think these studies are important to lay the foundation for future mechanistic studies. There are several areas I suggest the authors revise or tone down to not overstate what they did not show.

1. The study is framed in the context that asymmetric segregation of cellular components regulates the fate and behavior of somatic stem cells. While this group showed this in their previous papers, this study did not directly examine this. What the authors did is generate the tools to examine the establishment of an ER membrane diffusion barrier, show this is sensitive to photobleaching and Progerin overexpression, and correlates with asymmetric segregation of ubiquitinated proteins. What the authors did not do is show this is dependent on Lamin-A (overexpression of Progerin is not the same thing as knockdown or knockout of Lamin-A). The authors also did not track the fate of the cells that inherit the ubiquitinated proteins, in the presence of Progerin overexpression. Therefore, the authors should be encouraged to be more precise about their conclusions. Or they should consider performing a knockout/knockdown study of nuclear lamins if they want to claim that human ER barrier strength is dependent on lamins.
2. One of the other major conclusions is there is an asymmetric segregation of damaged proteins. Yet the study only tests mono- and poly-ubiquitinated proteins. Are all damaged proteins poly-ubiquitinated? Are all poly-ubiquitinated proteins damaged proteins? There should be more background and context here, and perhaps additional examples to strengthen this conclusion.
3. The authors test the establishment of an ER membrane diffusion barrier in 3D human forebrain organoids. They conclude there is an ER membrane diffusion barrier in human neural

© 2022. Published by The Company of Biologists under the terms of the Creative Commons Attribution License (https://creativecommons.org/licenses/by/4.0/).

progenitors, and this is a mechanism for establishing asymmetry. While the data showing the establishment of the ER membrane diffusion barrier is reasonable, there is data showing this barrier is required for asymmetric cell divisions is unconvincing. The authors should perform lineage tracing or some other experiment to provide support for this or tone down this conclusion.

Minor:
In Figure 1B legend, Lum-GFP is left and MemER-GFP is right.

First revision

Author response to reviewers' comments

Summary:

We are thankful for the constructive comments of the reviewers. The reviewers state that the “study is is well done and the results are clean” (Rev#1) and that our “work appears to be technically sound with use of solid assays and methodology and appropriate controls” (Rev#2). However, both reviewers question the novelty with regards to previous work that had been done in mouse progenitors. We understand that concern. However, we do believe that our extensive data using different types of progenitor cells (hESC and iPSC-derived) and intact brain organoid imaging clearly demonstrate the establishment of an ER-diffusion barrier in human cells. We believe that this is of fundamental importance for future research and that indeed the work presented here will be the foundation for future experiments to understand the molecular mechanisms and cellular consequences of an ER-diffusion barrier not only in mice but also in the context of human asymmetric cell divisions.
In a revised manuscript we will add novel data (e.g., characterization of used hESCs and iPSCs) and will carefully edit our manuscript to address the concerns previously raised by the reviewers.

Reviewer 1

“Overall the study is well done and the results are clean. My main criticism is the limited advances that it provides. It is expected that the findings made in mouse neuronal progenitors, for such a fundamental process, will be confirmed in human neuronal progenitors. While this constitutes a nice confirmation, it provides little advance to our understanding of asymmetric divisions. The authors could take advantage of their system to perform a more quantitative study on this barrier in a more progenitor-type specific manner.”

We thank the reviewer for their comments. We understand that the reviewer argues that our study does not go beyond what has been previously shown in yeast, C. elegans and mouse neural progenitor cells. However, we do feel that our study represents a significant advance on previous studies because we show that an ER-diffusion barrier-associated mechanism causing asymmetry is conserved also in human cells. The reviewer states “it is expected…for such a fundamental process…”. We would argue that a mere “expectation” is fundamentally distinct from “experimental confirmation”: which is what we aimed for in the current study. We are therefore convinced that our study represents a starting point for more detailed and mechanistic future studies. We will add these considerations to a revised manuscript, highlighting the relevance of the data we provide here in terms of human asymmetric cell division and neural development.
We have now more explicitly addressed this point in our revised manuscript as follows (page 10): “Given previous work in budding yeast, C. elegans and mouse progenitor cells, our finding that human progenitors establish an ER diffusion barrier is not unexpected. However, we here provide direct experimental evidence of an ER diffusion barrier in human progenitors using different lines of pluripotent cell-derived NPCs and brain organoids combined with FLIP imaging. Thus, the data shown here represent the foundation for future experiments with the aim to further our understanding of the role of asymmetric segregation during human progenitor cell divisions.”

© 2022. Published by The Company of Biologists under the terms of the Creative Commons Attribution License (https://creativecommons.org/licenses/by/4.0/).

“Major point:
- There is no data showing that the authors are actually looking at neuronal progenitors, and also there is no information on the nature of this progenitors. Are we looking at neuroectoderm, radial glia or other types of progenitors?”

We apologize that we had not more explicitly discussed previous characterizations of the distinct types of progenitors we had used. The used iPSC- and ESC-derived progenitor cells were characterized before, analyzing their potency and behavior (e.g., Costa et al., 2016 Cell Reports for hESCs and Hruska-Plochan et al., 2021 BioRxiv for iPSCs). Further, we have now included in our revised manuscript novel experimental data using a set of molecular marker proteins (e.g., SOX2, NESTIN, PLZF) to characterize the used iPSC- and ESC-derived progenitor cells. Please refer to modified Figure S1 and modified text.
Page 5:
“First, we developed tools to visualize the human ER membrane and lumen in human NPCs, derived from human embryonic stem cells (hESC) and expressing markers of NPCs such as SOX2, NESTIN, PLZF (Figure S1A) (Costa et al., 2016)”
Page 6:
“Again, we found that iPSC-derived NPCs, expressing SOX2, NESTIN, PLZF, established a diffusion barrier in the ER membrane during anaphase (Figure S1B-D).”

“Minor point:
- Some references cited in the text do not appear in the reference list (for example bin Imtiaz et al. 2021)”

We apologize for this mistake and have carefully checked the revised manuscript for proper citations in the text and reference list.

Reviewer 2

“The work appears to be technically sound with use of solid assays and methodology and appropriate controls. However, the work is correlative and does not show a causal relationship between the establishment of the ER diffusion barrier and the asymmetric segregation of damaged proteins, underlying the specification of fate and behavior of human neural progenitor cells. With that said, I think these studies are important to lay the foundation for future mechanistic studies. There are several areas I suggest the authors revise or tone down to not overstate what they did not show.”

We thank the reviewer for their thoughtful and constructive criticisms/suggestions.

“1.The study is framed in the context that asymmetric segregation of cellular components regulates the fate and behavior of somatic stem cells. While this group showed this in their previous papers, this study did not directly examine this. What the authors did is generate the tools to examine the establishment of an ER membrane diffusion barrier, show this is sensitive to photobleaching and Progerin overexpression, and correlates with asymmetric segregation of ubiquitinated proteins. What the authors did not do is show this is dependent on Lamin-A (overexpression of Progerin is not the same thing as knockdown or knockout of Lamin-A). The authors also did not track the fate of the cells that inherit the ubiquitinated proteins, in the presence of Progerin overexpression. Therefore, the authors should be encouraged to be more precise about their conclusions. Or they should consider performing a knockout/knockdown study of nuclear lamins if they want to claim that human ER barrier strength is dependent on lamins.”

We understand the reviewer’s concern and feel that their comment is a valid criticism of our work. We agree with the reviewer that our interpretations were partially imprecise (we have now carefully avoid unsubstantiated claims regarding a potential role for Lamin-A, page 7) and rephrased above-mentioned sections. We understand that tracking cell fate would be an exciting experiment; however, this is an extremely challenging experiment and currently not feasible as this would require the establishment and validation of reporter-systems to visualize (or at least correlate) live cells with the inheritance of ubiquitinated proteins. Further, combining FLIP with lineage tracking is extremely difficult (indeed, we have tried but did not succeed; obviously, one

© 2022. Published by The Company of Biologists under the terms of the Creative Commons Attribution License (https://creativecommons.org/licenses/by/4.0/).

problem is that at least some of the cells after FLIP are extremely dim). We would love to include such experiments but this is technically at this time extremely challenging (and close to impossible). Therefore, we followed the reviewer's advice and have been much "precise about their conclusions". For changes please refer to page 9:
"Thus, our results identify a diffusion barrier in the ER membrane established in human NPCs during cell divisions." (We deleted statement that this is "required" for asymmetric cell division).
"However, at this time it remains unknown if the ER diffusion barrier is indeed required for proper asymmetric cell divisions. Furthermore, the fate of cells establishing a barrier in the ER membrane will have to be analyzed in future experiments, given the current technical difficulty to combine FLIP approaches with imaging-based lineage tracing in human ESC-derived brain organoids."

"2.One of the other major conclusions is there is an asymmetric segregation of damaged proteins. Yet the study only tests mono- and poly-ubiquitinated proteins. Are all damaged proteins poly-ubiquitinated? Are all poly-ubiquitinated proteins damaged proteins? There should be more background and context here, and perhaps additional examples to strengthen this conclusion."

Again, we agree with the reviewer that our initial version was oversimplifying the biology of mono- and poly-ubiquitinated proteins. Indeed, ubiquitination of proteins does label damaged proteins but certainly also detects proteins with an ubiquitin-mediated post-translational modification that is involved in multiple different pathways independent of damage. Thus, we now more extensively discuss the relevance of mono- and poly-ubiquitinated proteins in the context of progenitor cell divisions in the revised manuscript (please refer to page 7). We have now carefully edited the manuscript to avoid oversimplifications and non-justified conclusions in the context of segregation of damaged proteins during neural progenitor cell divisions.
Page 7:
"Previous studies suggested a correlation between the strength of the ER membrane diffusion barrier and asymmetric segregation of mono- and poly-ubiquitinated proteins, as an indirect measure of levels of damaged proteins, even though ubiquitination is also involved in a plethora of biological processes independent of protein damage (Komander and Rape, 2012; Clay et al., 2014; Moore et al., 2015)"

"3.The authors test the establishment of an ER membrane diffusion barrier in 3D human forebrain organoids. They conclude there is an ER membrane diffusion barrier in human neural progenitors, and this is a mechanism for establishing asymmetry. While the data showing the establishment of the ER membrane diffusion barrier is reasonable, there is data showing this barrier is required for asymmetric cell divisions is unconvincing. The authors should perform lineage tracing or some other experiment to provide support for this or tone down this conclusion."

We understand that this is a limitation of our work. However, the experiments suggested are extremely complicated as the ability to perform lineage tracing in 3D human forebrain organoids following FLIP is extremely limited. Indeed, there is - to our knowledge – only rather limited data of lineage tracing in intact human brain organoids on a single cell level (an indeed, each single progenitor cell lineage, e.g., clone, would require the use of a single organoid). Thus, doing "just" lineage tracing is very challenging. Then to combine this with FLIP experiments (which are by themselves extremely challenging) is almost impossible (see above). We agree that future experiments will need to address how an ER-diffusion barrier affects asymmetric cell division in human neurogenic cells. However, we do believe that our study, using mosaic genome-edited human brain organoids combined with FLIP imaging in intact organoids, does indeed go beyond the state of the art. We will highlight the technical limitations that make it – at this time – extremely challenging to combine FLIP with lineage tracing experiments. Furthermore, we will follow the reviewers advice and will "tone down this conclusion". For changes please refer, for example, to the following changes:
Page 9 (see above):
"Thus, our results identify a diffusion barrier in the ER membrane established in human NPCs during cell divisions." (We deleted statement that this is "required" for asymmetric cell division).
"However, at this time it remains unknown if the ER diffusion barrier is indeed required for proper asymmetric cell divisions. Furthermore, the fate of cells establishing a barrier in the ER membrane will have to be analyzed in future experiments, given the current technical difficulty to combine FLIP approaches with imaging-based lineage tracing in human ESC-derived brain organoids."

© 2022. Published by The Company of Biologists under the terms of the Creative Commons Attribution License (https://creativecommons.org/licenses/by/4.0/).

"Minor:
In Figure 1B legend, Lum-GFP is left and MemER-GFP is right."

We apologize for this mistake that has been corrected.

Second decision letter

MS ID#: DEVELOP/2022/200613

MS TITLE: Human neural progenitors establish a diffusion barrier in the ER membrane during cell division

AUTHORS: Khadeesh Bin Imtiaz, Lars N Royall, Daniel Gonzalez-Bohorquez, and Sebastian Jessberger
ARTICLE TYPE: Research Report

I am happy to tell you that your manuscript has been accepted for publication in Development, pending our standard ethics checks.

Reviewer 2

Advance summary and potential significance to field

This paper uses advanced microscopy methods to demonstrate the establishment of a diffusion barrier during cellular division of human neural progenitors. While the presence of a diffusion barrier has been well established in model organisms, i.e., budding yeast and C. elegans, this paper takes the first step to validating a model using human forebrain organoids which could lead to future studies of the mechanisms by which human neural progenitors perform asymmetric segregation of cellular components.

Comments for the author

The authors have satisfactorily addressed my concerns in the revised manuscript. The additional discussion of the limitations and challenges of doing lineage tracing in the human brain organoid system is important for the field to know and is also much appreciated.

© 2022. Published by The Company of Biologists under the terms of the Creative Commons Attribution License (https://creativecommons.org/licenses/by/4.0/).
